# A Novel Cellular Therapy to Treat Pancreatic Pain in Experimental Chronic Pancreatitis Using Human Alpha-1 Antitrypsin Overexpressing Mesenchymal Stromal Cells

**DOI:** 10.3390/biomedicines9111695

**Published:** 2021-11-16

**Authors:** Rebecca P. Chow, Kevin Nguyen, Wenyu Gou, Erica Green, Katherine Morgan, William Lancaster, Kristi Helke, Charlie Strange, Hongjun Wang

**Affiliations:** 1 Department of Surgery, Medical University of South Carolina, Charleston, SC 29425, USA; pyrc201@gmail.com (R.P.C.); nguyenlu@musc.edu (K.N.); gou@musc.edu (W.G.); greeeric@musc.edu (E.G.); morganka@musc.edu (K.M.); lancastw@musc.edu (W.L.); 2 Department of Comparative Medicine, Medical University of South Carolina, Charleston, SC 29425, USA; helke@musc.edu; 3 Department of Medicine, Medical University of South Carolina, Charleston, SC 29425, USA; strangec@musc.edu; 4 Ralph H. Johnson VA Medical Center, Charleston, SC 29425, USA; 5 Center for Cellular Therapy, Medical University of South Carolina, Charleston, SC 29425, USA

**Keywords:** mesenchymal stromal cells, chronic pancreatitis, pain, inflammation, TRPV1, mast cells, antitrypsin

## Abstract

Chronic pancreatitis (CP) is characterized by pancreatic inflammation, fibrosis, and abdominal pain that is challenging to treat. Mesenchymal stromal cells (MSCs) overexpressing human alpha-1 antitrypsin (hAAT-MSCs) showed improved mobility and protective functions over native MSCs in nonobese diabetic mice. We investigated whether hAAT-MSCs could mitigate CP and its associated pain using trinitrobenzene sulfonic acid (TNBS)-induced CP mouse models. CP mice were given native human MSCs or hAAT-MSCs (0.5 × 10^6^ cells/mouse, i.v., *n* = 6–8/group). The index of visceral pain was measured by graduated von Frey filaments. Pancreatic morphology and pancreatic mast cell count were analyzed by morphological stains. Nociceptor transient receptor potential vanilloid 1 (TRPV1) expression in dorsal root ganglia (DRG) was determined by immunohistochemistry. hAAT-MSC-treated CP mice best preserved pancreatic morphology and histology. MSC or hAAT-MSC infusion reduced abdominal pain sensitivities. hAAT-MSC therapy also suppressed TRPV1 expression in DRG and reduced pancreatic mast cell density induced by TNBS. Overall, hAAT-MSCs reduced pain and mitigated pancreatic inflammation in CP equal to MSCs with a trend toward a higher pancreatic weight and better pain relief in the hAAT-MSC group compared to the MSC group. Both MSCs and hAAT-MSCs might be used as a novel therapeutic tool for CP-related pain.

## 1. Introduction

Chronic pancreatitis (CP) is a progressive condition in the pancreas that leads to significant impairment of both endocrine and exocrine functions [1]. Its global prevalence and incidence are estimated at 76.2 and 20.6 per 100,000 people, respectively [2]. CP is characterized by unrelenting abdominal pain, persistent pancreatic inflammation, and irreversible morphological changes such as fibrosis of the pancreas [3]. Up to 90% of patients suffer from abdominal pain [4], and there are only limited therapeutic options for pain relief [5]. The etiology of pain is poorly understood and probably multifaceted with alternations in peripheral nociception, peripheral/pancreatic neuropathy, and altered neuroplasticity [6,7,8]. The pain is thought to be a result of neurogenic and pancreatic inflammation [4]. Current medical therapy in CP pain control uses a stepped escalation approach that often leads to opioid dependence [5]. The adverse effects of narcotics and complications of interventional therapies may account for a substantial morbidity in patients [9]. Thorough understanding of the origin of pain in CP will be essential to identify novel therapeutic interventions that are urgently needed.

Mesenchymal stromal cells (MSCs) are fibroblast-like adult stem cells that possess the ability to self-renew. They have a multi-lineage differentiation potential and are recognized by unique cell surface markers [10]. The richest sources of MSC are adipose tissue and bone marrow, but they can also be found in umbilical cord, placenta, synovial fluid, and other sources [11]. MSCs are being studied in a variety of diseases because of their immunoregulatory abilities, antifibrotic effects, and tissue protective features. Convincing evidence from rodent studies and clinical trials shows that MSCs reduce chronic and neurological pain associated with knee osteoarthritis, critical limb ischemia, neuropathy, and diabetic neuropathy [12,13,14,15]. In a rodent study, a single injection of bone marrow-derived MSCs (BM-MSCs) reversed pain hypersensitivity in rats after injury, and this effect lasted for at least 22 weeks [16]. Moreover, BM-MSCs alleviated neuropathic pain in the early stages of streptozotocin-induced rat diabetes [15]. In rat models of pancreatitis, MSCs inhibited inflammation and pancreatic damage [17,18], supporting the potential use of MSCs as a regenerative cellular therapy for CP patients.

Alpha-1 antitrypsin (AAT) is a circulating protein primarily synthesized in the liver. It is an acute phase protein and a natural serine proteases inhibitor [19]. It plays a key role in reducing inflammation by suppressing proinflammatory cytokine production in whole blood [20], inhibiting neutrophil superoxide production [21] and protease activities [22], preventing cell apoptosis [23,24], and stimulating cell growth and proliferation [19,25]. AAT expression in human peripheral blood mononuclear cells (PBMCs) is modulated by promoter methylation of an inducible AAT coding gene SERine Protein INhibitor-A1 (SERPINA1) [26]. Because AAT therapy has shown beneficial effects in inflammation-related disease models for rheumatoid arthritis, lupus, and type 1 diabetes (T1D) [27,28], we overexpressed human AAT (hAAT) in MSCs to show improved intrinsic properties and sustained efficacy in mice with T1D [28]. hAAT-MSCs reduced immune cell infiltration into pancreatic islets, and one injection of hAAT-MSCs delayed the onset of T1D longer than native MSCs in a NOD mouse model [28]. Together, hAAT-MSCs may possess improved therapeutic effects compared to native MSCs in the treatment of CP pain.

In this study, we compared the hypoalgesic and therapeutic effects of hAAT-MSCs and MSCs in CP. We used a painful CP mouse model that was previously established [29,30] by injecting trinitrobenzene sulfonic acid (TNBS) into the bile duct of healthy mice. After confirmation of the presence of CP features one week after TNBS infusion, we injected native MSCs or hAAT-MSCs via the tail vein of some of the TNBS mice. We compared the morphology and histology of pancreases from vehicle controls (PBS infusion via bile duct), TNBS mice without MSC treatment (TNBS), and TNBS mice with native MSCs (TNBS + MSCs) or hAAT-MSCs (TNBS + hAAT-MSCs). We also compared the index of visceral pain, expression of transient receptor potential cation channel subfamily V member 1 (TRPV1) in dorsal root ganglion (DRG), and pancreatic mast cell density among the four groups.

## 2. Materials and Methods

### 2.1. Animal

A total of 56 male C57BL/6 mice at 10–12 weeks of age (The Jackson Laboratory, Bar Harbor, ME, USA) were used in this study. CP was induced by a single infusion of 0.4% TNBS (50 µL; Sigma-Aldrich, St. Louis, MO, USA) via bile duct as previously described [29]. Mice were housed and cared for under standard operating procedures of the Animal Facility at the Ralph H. Johnson VA Medical Center (VAMC) in Charleston, SC, USA. All study procedures were approved by the Institutional Animal Care and Use Committee of the VAMC (Protocol #653, Date of issue: 28 May 2019, Expiration date: 27 May 2022).

The application of TNBS resulted in a model that showed progression from acute pancreatitis to CP [30]. TNBS rodent models have been shown to resemble CP morphological features in human that include fibrosis, inflammation, monocyte infiltration, fatty replacement, atrophy, and related pain behaviors [30,31,32]. Compared to other CP models, the TNBS model is a simple single injection via the bile duct, while other models usually require multiple doses injections (i.e., caerulein + ethanol model) or application of ethanol to the drinking water (i.e., Dibutyltin chloride model) [31,33,34,35].

### 2.2. hAAT-MSC and MSC Preparation and Infusion

Human MSCs were isolated from bone marrow specimens of healthy donors purchased from American Type Culture Collection (ATCC, Old Town Manassas, VA, USA). Cells were cultured in Dulbecco’s modified Eagle’s medium (DMEM), supplemented with 10% fetal bovine serum (FBS), 1% penicillin and streptomycin in 5% CO_2_ at 37 °C. hAAT-MSCs were prepared by lentivirus infection of native MSCs as previously described [28]. Briefly, MSCs were transduced with the lentiviral vector encoding hAAT and green fluorescent protein (GFP) [36]. Presence of GFP^+^ cells was detected under a fluorescent microscope 96 h after viral infection. hAAT-MSCs and native MSCs at passage 5–8 (0.5 × 10^6^ cells per mouse) were infused into the mice via tail veins one week after TNBS treatment.

### 2.3. Histological Scoring of the Pancreas

Pancreas tissues were embedded in paraffin, sectioned (5 μm), and stained with hematoxylin and eosin (H&E) and Masson’s Trichrome. Sections were deparaffinized in xylene, dehydrated in alcohol gradients. H&E staining and Masson’s Trichrome staining were performed by the Histology and Immunohistochemistry Laboratory at the Medical University of South Caroline (Charleston, SC, USA). The sections were then examined by a pathologist blinded to treatment group. The severity of pancreatitis was scored for in six categories [37]:

(1) total area preserved (0%—the whole pancreas is damaged to 100%—no damage in the pancreas)
(2) inflammation (0—no inflammation; 1—less than 25% inflammation; 2—25–50% inflammation; 3—50–75% inflammation and 4–100% inflammation)
(3) necrosis (0—no necrosis; 1—minimal necrosis; 2—mild necrosis; 3—moderate necrosis; 4—severe necrosis)
(4) fibrosis (0—no fibrosis; 1—minimal fibrosis; 2—mild fibrosis; 3—moderate fibrosis; 4—severe fibrosis)
(5) vacuole formation in acinar cells (0—no vacuolization; 1—less than 25% vacuolization; 2—25–50% vacuolization; 3—50–75% vacuolization and 4—100% vacuolization)
(6) interlobular edema (0—no interlobular edema; 1—minimal interlobular edema; 2—mild interlobular edema; 3—moderate interlobular edema; 4—severe interlobular edema)

The total area preserved was scored according to the histoarchitectural features of the whole slide, while the other five categories were scored in five fields/sections. The results of each category, excluding the total area preserved, were expressed as the sum of those five fields.

### 2.4. May-Grünwald-Giemsa Staining for Detection of Mast Cells

Paraffin-embedded pancreatic tissues from 3–4 mice were stained using May-Grünwald-Giemsa staining [38,39] according to the manufacturer’s protocol (Eng Scientific, Clifton, NJ, USA). In brief, sections were deparaffinized and incubated with May-Grünwald Solution for 25 min follow by two washes with PBS. The sections then incubated with Giemsa Stain for 20 min, washed with PBS, dehydrated in ethanol, and cleared with xylene. Mast cells were counted in 10 fields/section under a light microscope (Olympus BX40). Researchers performing cell counts remained blinded to the treatment group. Results are expressed as mast cell/mm^2^.

### 2.5. Immunohistochemistry

T9-12 DRG from mice were removed as previously described [40] and placed in 4% paraformaldehyde for fixation. Fixed DRG were then placed in 20% sucrose for cryoprotection, embedded in optimal cutting temperature compound (Sakura Finetek, Torrance, CA, USA), frozen, and sectioned at 14 μm using a cryostat. DRG sections were incubated with rabbit anti- TRPV1 antibody (1:100; # PA5-77317, Invitrogen, Waltham, MA, USA) and then with Alexa Fluor 568 goat anti-rabbit IgG (1:500; #A-11011, Invitrogen). Slides were observed using a Zeiss AxioImager M2 fluorescence microscope. Intensity was calculated using ImageJ software.

### 2.6. Behavioral Assessment of Pain

Von Frey filament (vFF) probing of the paw and abdomen was used to assess pain postoperatively. VFF probing has been used and established as a measure of referred abdominal mechanical hypersensitivity [30,32,41,42]. Starting from one week after MSC injection, mice were acclimated for 1 h in plastic cylinders with a mesh floor before testing. Paw and abdominal mechanical referred pain were measured by the application of calibrated vFFs (North Coast Medical, Morgan Hill, CA, USA) with increasing applied forces to the paw and upper abdominal area five times each for 1–2 s. A response was considered positive when the mouse raised, retracted, or licked its abdomen (withdrawal response). Data are expressed as the lowest calibrated vFF applied forces needed to trigger a positive response per mouse per experimental group.

### 2.7. Statistical Analyses

Data are expressed as mean ± standard error of the mean (SEM). Differences between groups were analyzed by one-way ANOVA with Tukey post hoc test by GraphPad Prism 9.

## 3. Results

### 3.1. Mice Treated with MSCs or hAAT-MSCs Show Less Pancreatic Injury after TNBS Infusion

**Figure 1:**
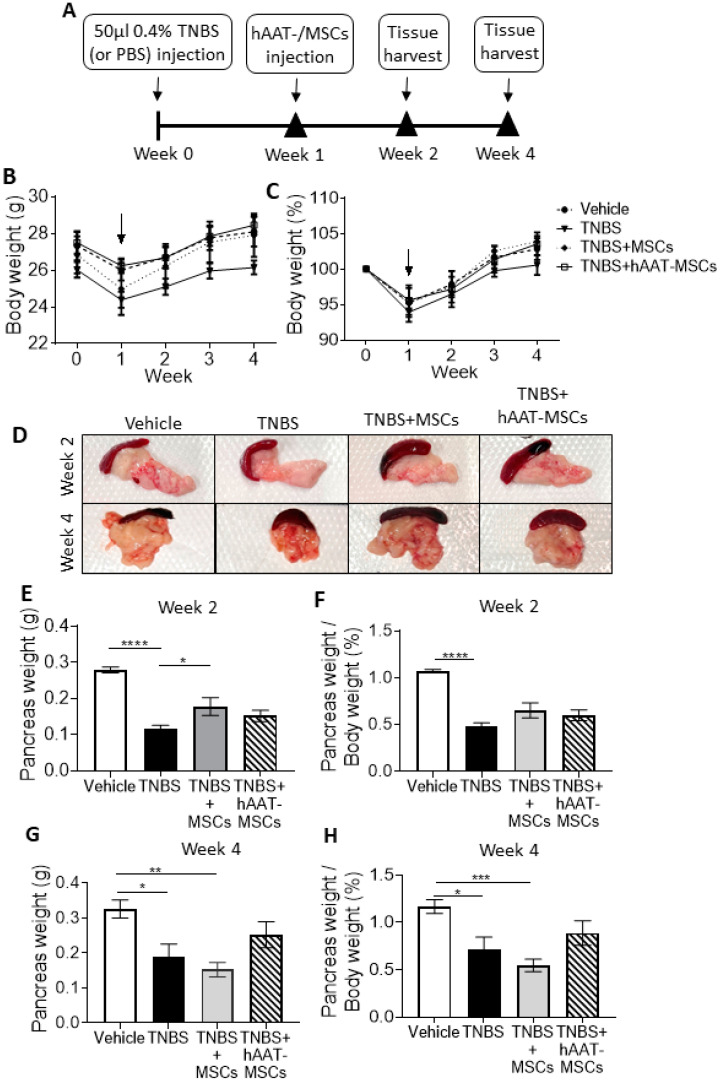
The effects of mesenchymal stromal cell (MSC) therapy on the body weight and pancreas weight of chronic pancreatitis (CP) mice. (**A**) Schematic diagram of CP induction by trinitrobenzene sulfonic acid (TNBS), cell infusion, and tissue collection and analysis. (**B**,**C**) Body weight changes represented in total body weight and percentage change. Arrow indicates the infusion of human alpha-1 antitrypsin-overexpressed mesenchymal stromal cells (hAAT-MSCs) or native MSCs (Vehicle = 6, TNBS = 8, TNBS + MSCs = 6, TNBS + hAAT-MSCs = 6 mice per group). (**D**) Images of pancreases collected from Vehicle, TNBS mice, or TNBS mice treated with MSCs or hAAT-MSCs 0.5 × 10^6^ cells/mouse). (**E**–**H**) Pancreas weights and percent ratio of pancreas weight to body weight at (**E**–**F**) 2 week-(Vehicle = 7, TNBS = 8, TNBS + MSCs = 8, TNBS + hAAT-MSCs = 7 mice per group), and (**G**,**H**) 4 week post TNBS treatment. Scale bar = 1 cm. Data presented are mean ± SEM. One-way ANOVA and Tukey’s test were used. * *p* < 0.05, ** *p* < 0.01, *** *p* < 0.001, **** *p* < 0.0001.

We induced CP using bile duct infusion of TNBS, a nitroaryl oxidizing acid that can cause oxidative stress, pancreas damage, and pain [30,43] in C57BL/6 mice (Figure 1A). One week after TNBS injection, mice were divided into three groups: TNBS (CP control), TNBS + MSCs (CP mice receiving native MSCs), and TNBS + hAAT-MSCs (CP mice receiving hAAT-MSCs). In the TNBS + MSC and TNBS + hAAT-MSC groups, each mouse received a single dose of 0.5 × 10^6^ cells via tail vein injection (Figure 1A). Mice receiving PBS infusion at week zero were used as healthy controls (vehicle). At one week after TNBS infusion, CP mice had a noticeable reduction in bodyweight compared to the vehicle. CP mice also showed a slower recovery rate compared to other groups in terms of body weight (Figure 1B) and percentage change of body weight (Figure 1C). The pancreas size and weight in TNBS mice were significantly lower compared to the vehicle group at weeks two and four (Figure 1D–F). hAAT-MSC-treated mice had the heaviest pancreas weight and highest ratio of pancreas weight to body weight at week four compared to CP controls (Figure 1G,H).

### 3.2. MSC Infusion Preserves Pancreatic Histology in CP Mice

**Figure 2:**
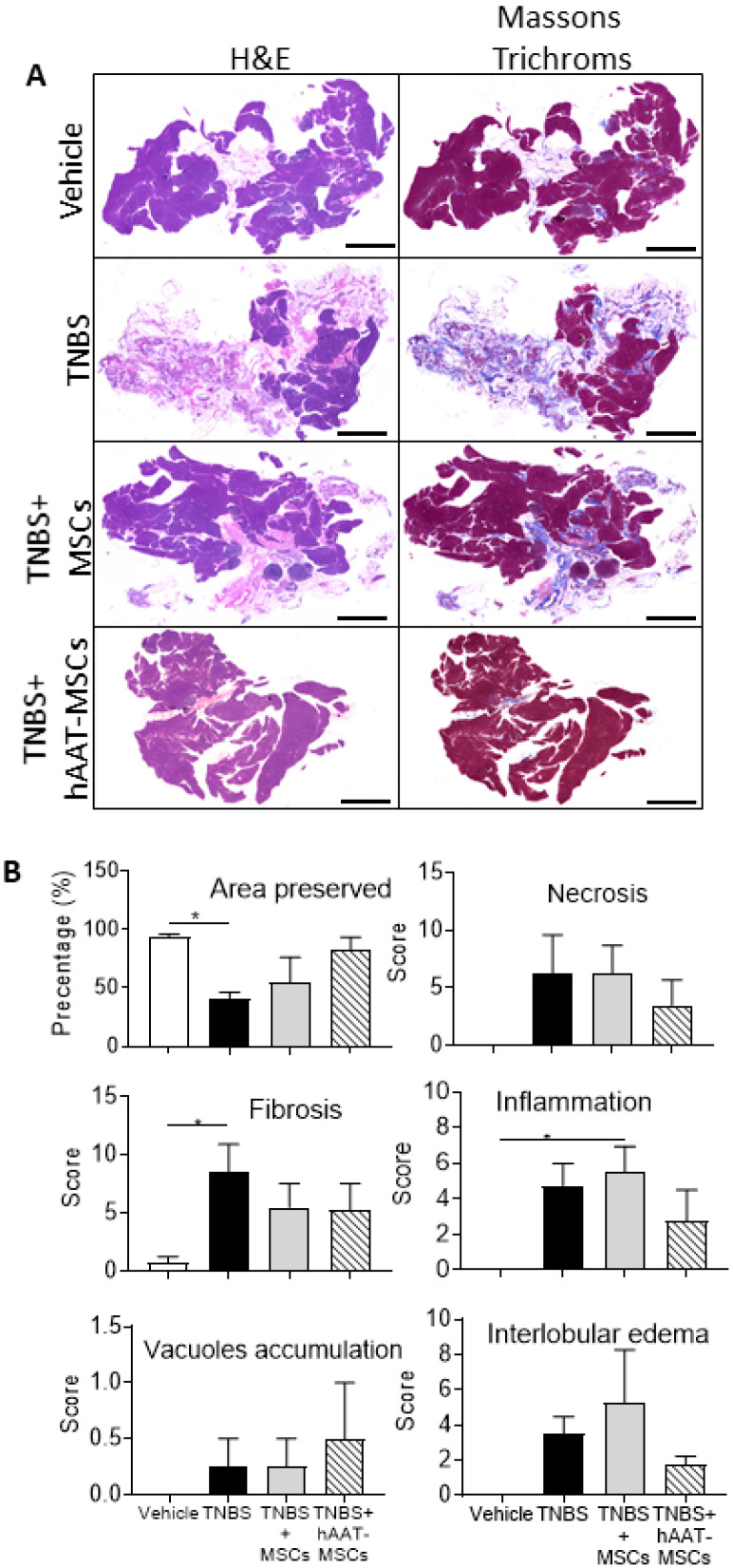
Human alpha-1 antitrypsin-overexpressed mesenchymal stromal cells (hAAT-MSC) and MSC infusions improve pancreas morphology of chronic pancreatitis (CP) mice 4 weeks post trinitrobenzene sulfonic acid (TNBS) treatment. To assess whether MSCs have any protective effects on pancreas damage, pancreas tissues were stained with Hematoxylin and Eosin (H&E) and Masson Trichrome. (**A**) Pancreatic structure of hAAT-MSC or native MSC-treated TNBS mice were better preserved and showed significantly reduced staining of collagen and more normal histology. (**B**) The stained pancreatic sections were scored by a pathologist according to six categories (*n* = 4 mice in each group). Increased fibrosis and inflammation were seen in the pancreas of TNBS-treated mice compared to that of the vehicle, while the pancreas in those treated with native MSCs or hAAT-MSCs displayed better morphology. This data further confirmed the protective effects of MSCs on TNBS-induced pancreas damage. Scale bar = 2 mm. Data presented are mean ± SEM. One-way ANOVA and Tukey’s test were used. * *p* < 0.05.

Bile duct infusion of TNBS consistently generated a mouse model that resembles the CP features often seen in humans (Figure 2A). At weeks two and four, the pancreases of TNBS mice demonstrated a significant loss of pancreatic structure and large regions of intralobular fibrosis (Figure 2A,B and Figure S1). In contrast, the pancreases of mice treated with native MSCs or hAAT-MSCs were better preserved with significantly reduced fibrosis-positive staining (Figure 2A,B). Blinded histological evaluation of these slides by an independent pathologist further confirmed increased fibrosis, interlobular edema, and inflammation in the pancreases of TNBS mice compared to the healthy controls. Furthermore, mice receiving native MSCs or hAAT-MSCs had much more of a preserved area of the pancreas (Figure 2B). Mice receiving hAAT-MSCs also showed a trend of reduced inflammation, necrosis, fibrosis, and interlobular edema (Figure 2B), which might have contributed to better preserved pancreases.

### 3.3. MSC Infusion Ameliorates CP Pain

**Figure 3:**
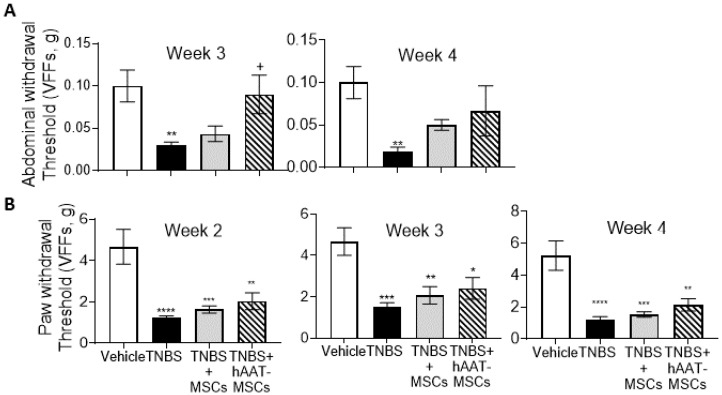
Human alpha-1 antitrypsin overexpressing mesenchymal stromal cells (hAAT-MSC) and MSC infusion reduced mechanical abdominal and paw sensitivities in trinitrobenzene sulfonic acid (TNBS) mice. Mechanical sensitivity was measured with Von Frey filaments (Vehicle = 6, TNBS = 8, TNBS + MSCs = 6, TNBS + hAAT-MSCs = 6 mice per group). TNBS-induced chronic pancreatitis (CP) reduced mechanical (**A**) abdominal and (**B**) paw sensitivities. MSC infusion showed reduced sensitivity to the stimulations, and hAAT-MSCs had a numerically larger although not statistically significant effect on this reduction compared to native MSCs (*p* = not significant). The data suggest that MSC infusion may relieve pain in CP. Data presented are mean ± SEM. One-way ANOVA and Tukey’s test were used. + *p* < 0.5 vs. TNBS, * *p* < 0.05, ** *p* < 0.01, *** *p* < 0.001, **** *p* < 0.0001 vs. Vehicle.

One of the hallmarks of CP is sustained visceral pain that often radiates to other body parts. TNBS injection caused pain in mice that mimics the clinical symptoms of CP pain as reflected by the surrogate measurements of paw and abdomen sensitivity using the graduated vFF referred as mechanical hypersensitivities or pain threshold as reported previously [29,30]. In this study, TNBS mice showed a significant increase in abdominal and paw withdrawal sensitivities throughout the experiment compared to the healthy controls (Figure 3A,B). Both native MSC and hAAT-MSC infusions reduced sensitivity to these stimulations in treated TNBS mice. hAAT-MSC-treated mice showed significantly reduced abdominal sensitivity compared to mice treated with TNBS at week three. At week four, a trend of reduced abdominal sensitivity was observed in TNBS mice treated with MSCs, but the differences did not reach significance. This data suggests that hAAT-MSCs might have a more pronounced effect on relieving CP pain compared to native MSCs.

### 3.4. MSC Pain Reduction Is Associated with Downregulation of TRPV1 Expression

**Figure 4:**
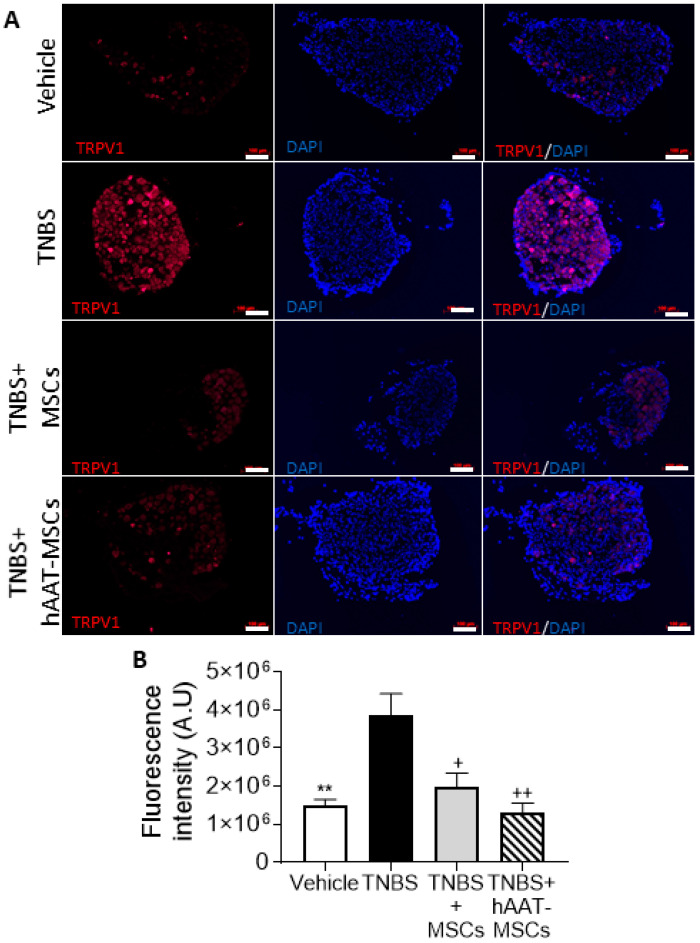
Human alpha-1 antitrypsin-overexpressed mesenchymal stromal cells (hAAT-MSC) and MSC infusions reduced transient receptor potential cation channel subfamily V member 1 (TRPV1) expression. Dorsal root ganglions (DRG) from T9-12 were harvested at 4 weeks after trinitrobenzene sulfonic acid (TNBS) injection and (**A**) the expression of TRPV1, a pain receptor that is associated with chronic pain, was examined (Vehicle = 5, TNBS = 5, TNBS + MSCs = 3, TNBS + hAAT-MSCs = 3 mice per group). Scale bar = 100 μm (**B**) TRPV1 expression in DRGs increased significantly in TNBS-treated mice, and hAAT-MSC or MSC treatment drastically reduced its expression, suggesting that MSCs might reduce pain via downregulation of TRPV1 expression. Data presented are mean ± SEM. One-way ANOVA and Tukey’s test were used. ** *p* < 0.01 vs. Vehicle, + *p* < 0.05, ++ *p* < 0.01 vs. TNBS.

Neurogenic inflammation contributes to pain-related behaviors in CP. Pain and inflammation associated with pancreatitis has been shown to require transient receptor potential (TRP) channel TRPV1 [31,32,33]. To study the mechanism of MSCs in CP pain, we extracted DRG from T9-12 in mice at week four and stained them with an anti-TRPV1 antibody. Neuronal TRPV1 expression in TNBS mice increased significantly compared to healthy controls, concurring with previous studies [31,32] (Figure 4A). Both native MSCs and hAAT-MSCs drastically reduced TRPV1 expression in TNBS mice, with the numerical expression in the hAAT-MSC group being lower than that of the native MSC group (Figure 4B). The data suggests that MSC infusions may reduce pain by suppressing TRPV1 expression in the DRG of CP mice.

### 3.5. MSC Infusions Inhibit Mast Cell Infiltration into the Pancreas of CP Mice

**Figure 5:**
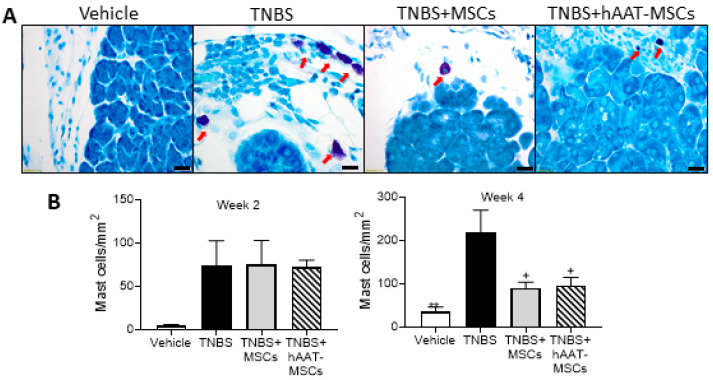
Pancreatic mast cell density is reduced in both groups of human alpha-1 antitrypsin-overexpressed mesenchymal stromal cells (hAAT-MSC) and MSC-treated mice compared to trinitrobenzene sulfonic acid (TNBS) mice. (**A**) Pancreatic sections were stained for mast cells using May-Grünwald–Giemsa stain (Vehicle = 3, TNBS = 3, TNBS + MSCs = 3 for week 4 and 4 for week 2, TNBS + hAAT-MSCs = 3 mice per group). Arrows indicated mast cells. Scale bars represent 20 μm. (**B**) Both groups of MSC mice had reduced mast cell density. Data are showed as mean ± SEM. One-way ANOVA and Tukey’s test were used. ** *p* < 0.01 vs. Vehicle, + *p* < 0.05 vs. TNBS.

Perineural mast cells are specifically enriched in neuropathic pain in CP [34]. To compare the presence of mast cells, pancreatic sections from all groups were stained with May–Grünwald–Giemsa staining. As is evident in Figure 5, TNBS mice had significantly increased mast cells density in the pancreas compared to the vehicle at four weeks after TNBS injection. MSC infusions did not reduce the mast cells density in TNBS mice at week two (Figure 5B). In contrast, at week four, mice treated with hAAT-MSCs or native MSCs showed significantly fewer mast cells in the pancreas compared to TNBS mice (Figure 5A,B), suggesting that the improvement in CP pain seen with MSCs is associated with reduced mast cell infiltration.

## 4. Discussion

Developing effective therapies for CP pain continues to be challenging. There are few mechanistic studies done regarding this disease and existing studies acknowledge the need for a better understanding of pain pathogenesis [4]. Based on our current knowledge, CP pain is a result of somatic and visceral pain derived from neurogenic and pancreatic inflammation [4]. Since MSCs have been shown to be a promising therapy for tissue inflammation in many models [10,44,45,46,47,48], we explored if MSCs might relieve the pain associated with CP. We also compared whether overexpression of hAAT in MSCs enhances their protective ability.

To examine the therapeutic effects of MSCs in vivo, we used a TNBS mouse model that resembles major CP features including pancreatic atrophy and pain in humans. It has been shown that CP features in rodents induced by TNBS at week 6 remained similar extent as those at week 3 [31]. We found synchrony between increased pancreatic inflammation, upregulation of TRPV1 expression in the DRG, and pancreatic mast cell infiltration following a TNBS injury. Intravenous MSC infusions improved all of these features and resulted in less pancreatic fibrosis at one month. hAAT-MSCs seemed to have a more profound protection based on preserved pancreas area and weight, reduced sensitivity to stimulation (pain), and reduced TRPV1 expression in the DRG of CP mice.

To date, there are only a few studies in which MSCs were administered for the treatment of CP. The source of MSCs included mouse adipose-derived MSCs [49,50], rat umbilical cord MSCs, human amnion-derived MSCs [18], and rat BM-MSCs [51,52]. Consistent with our data, all the studies showed that MSC infusion reduced pancreatic damage and decreased fibrosis. However, none of those studies investigated the mechanisms by which MSC infusions might impact CP pain. In this study, TNBS-induced CP pain was confirmed by vFF probing of the abdomen and paw. VFF probing is a well-established behavioral pain assay that is used as a surrogate marker for visceral pain. TNBS mice receiving MSCs developed reduced abdominal mechanical sensitivity, suggesting that MSCs alleviated force-dependent abdominal referred pain. The evidence of reduced referred hyperalgesia in the paw by MSCs was weaker than in the abdomen, indicating that the measured abdominal mechanical referred pain is specific and originated from the intra-abdominal area. Interestingly, although human studies are few in number, there appears to be no association between pancreas morphology and CP pain in humans [53]. Therefore, to better understand the relationship between the TNBS mouse model and human acute or chronic pancreatitis pain, human trials will be required.

One major cause of CP pain is known to be TRPV1 activation [54,55]. TRPV1 is a capsaicin receptor that modulates neuronal release of the inflammatory neuropeptide substance P (SP) and calcitonin gene-related peptide (CGRP) [55], both of which are involved in CP pain and are upregulated in TNBS rodent model [32]. TRPV1 also regulates the activation of transient receptor potential ankyrin-1 (TRPA1), which mediates neurogenic inflammation and inflammatory hyperalgesia [54]. A study demonstrated that capsazepine, a TRPV1 inhibitor, suppressed SP release in CP mice [56]. Another study highlighted the synergistic role of TRPV1 and TRPA1 in acute pancreatitis development [57]. Early intervention with TRPV1 and TRPA1 antagonists (within three weeks of the development of acute pancreatitis) attenuated the transition to and development of CP and downstream pain behaviors in mice [54].

A novel finding of our study is that MSC infusions greatly reduce the expression of TRPV1 in TNBS mice. MSCs are known for their homing ability to sites of inflammation to moderate the release of growth factors and cytokines at the desired site. We could not identify any native MSC or hAAT-MSCs in the DRG (data not shown), suggesting that MSCs might not act directly on the neurons. Instead, they might act peripherally by repairing neuroplasticity in the pancreas [58], or via the release of a secretome, which has been shown to greatly lessen neuroinflammation and restore inflammatory cytokine and signaling molecule balance in diabetic neuropathy [59,60].

We also showed firsthand that MSCs reduce mast cell density in CP mice. Mast cell activation and visceral pain have been correlated in irritable bowel syndrome, cystitis, complex regional pain syndromes, pancreatic cancer, and CP [61,62,63,64]. Perineural mast cells usually interact with the nerve fibers by releasing mediators such as nerve growth factors, histamine, and tryptase, leading to the release of SP and other neuropeptides [65]. Evidence showed that BM-MSCs suppress mast cell degranulation, TNF-α production, chemokinesis and chemotaxis in vivo and in vitro via upregulation of COX2 and that BM-MSCs were facilitated through the activation of E-type prostanoid receptor 4 on mast cells [66]. In myocardial infarction, mast cells promote proliferation and migration of BM-MSCs by suppressing their myogenic differentiation through the platelet derived growth factor pathway via downregulation of miR-145/miR-143 [67]. Taken together, native MSCs and hAAT-MSCs might exert their anti-inflammatory ability via inhibiting mast cell migration to the target site or degranulation after arrival. Further research must be done to understand the interplay between MSCs and mast cells.

We have previously shown that hAAT-MSCs have better migration ability, increased stemness, and other factors critical for MSC function compared to native MSCs [28]. We could not definitively prove that hAAT-MSC infusions were more effective in reversing TNBS pancreatitis or pain compared to native MSCs in this study although a strong trend of better protection was observed in the hAAT-MSC group compared to the native MSC group (Table S1). Overall, the trends in mast cell infiltration, TRPV1 signaling, and inflammatory scores suggest that overexpression of alpha-1 antitrypsin (AAT) were helpful to decreasing pancreatic inflammation.

The theory by which we embarked upon this project was that AAT is a potent inhibitor of serine proteases. AAT is anti-inflammatory; it suppresses cytokine production, complement activation, and immune cell infiltration [28]. Studies have found an imbalance of protease-to-protease inhibitor in patients with CP [9], and recurrent acute pancreatitis is described in case reports of genetic AAT deficiency. If it is true that MSCs migrate to an inflamed local environment, then the paracrine local release of AAT might be an effective cure for CP. If MSCs work through more distant effects when given intravenously and have a limited paracrine function, then AAT would be more systemically active. There have not been well executed studies of intravenous AAT in acute or chronic pancreatitis, but these findings suggest that more formal human studies should be done.

One limitation of our study was that we used vFF probing as the primarily index of pain. While vFF probing is a widely accepted method to measure mechanical sensitivity of the abdomen, it may not be the best surrogate of internal visceral sensation. Therefore, observing pancreatic electrical stimulation induced pain behaviors via intra-abdominal electrodes may be a preferred choice to evaluate the magnitude of pancreatitis pain [32].

BM-MSCs are the most frequently used MSC in clinical trials and this cell type has been approved by the Food and Drug Administration for acute graft-versus-host disease [68]. Their ease of culture and their ability to differentiate make them a promising tool for cell therapy. Clinical trials using BM-MSCs show safety but weak efficacy in lung diseases [68]. The most common way to administrate MSCs is by intravenous infusion in which the majority of the cells are trapped in the lungs before migration to the target sites [69,70,71,72,73]. However, clinical trials using MSCs for inflammatory bowel diseases demonstrated that MSCs are not only safe but also therapeutically relevant with durable effects in patients [74]. There is currently no clinical trial in acute or chronic pancreatitis. We were able to locate MSCs in the pancreas from this (data not shown) and our previous study [50] suggesting that MSCs have the ability to reach the pancreas, but the precise pathway of travel is still unclear. In line with our study, MSC therapy showed promising results in preclinical studies of CP [18,50,51,53]. Further clarification on the pathophysiology of the disease would allow better tailoring of MSC therapy.

## 5. Conclusions

We demonstrated a novel cellular therapeutic approach to reduce pain associated with CP. Native MSC or hAAT-MSC therapy simultaneously reduce TRPV1 expression and mast cell density in the pancreas. Further research is needed to understand the molecular mechanisms of how MSCs exert their effects on TRPV1 and mast cells.

## Data Availability

The data presented in this study are available on request from the corresponding author.

## Supplementary Materials

The following are available online at https://www.mdpi.com/article/10.3390/biomedicines9111695/s1, Figure S1: Hematoxylin and Eosin (H&E) and Masson-Goldner stains of pancreas 2 week post trinitrobenzene sulfonic acid (TNBS) treatment. Human alpha-1 antitrypsin-overexpressed mesenchymal stromal cells (hAAT-MSCs) or MSCs protective effects were noticeable at two weeks (one week after MSC infusions). Scale bar = 2 mm. Table S1: A summary table of the experiment outcomes. Human alpha-1 antitrypsin-overexpressed mesenchymal stromal cells (hAAT-MSCs) showed trends of better protective effects than native MSC although results did not reach significance.

## Abbreviations

DRG = dorsal root ganglion, human alpha-1 antitrypsin overexpressing mesenchymal stromal cells = hAAT-MSCs, TRPV1 = transient receptor potential vanilloid subfamily 1, TRPA1 = transient receptor potential cation channel, MSCs = mesenchymal stromal cells.
