# Supplementary material for: A Novel Cellular Therapy to Treat Pancreatic Pain in Experimental Chronic Pancreatitis Using Human Alpha-1 Antitrypsin Overexpressing Mesenchymal Stromal Cells
Source: Biomedicines. 2021 Nov 16;9(11):1695. doi: 10.3390/biomedicines9111695 (PMC8615652; doi:10.3390/biomedicines9111695)
Supplement: Supplementary file 1 [file biomedicines-09-01695-s001.zip › biomedicines-1441034-supplementary.pdf]

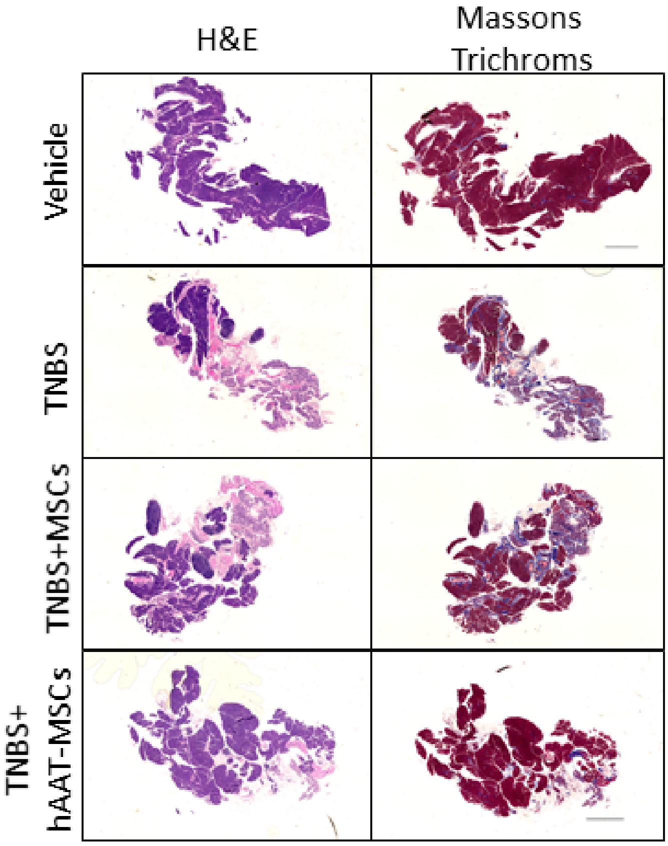

Figure S1: Hematoxylin and Eosin (H&E) and Masson-Goldner stains of pancreas 2 week post trinitrobenzene sulfonic acid (TNBS) treatment. Human alpha-1 antitrypsin-overexpressed mesenchymal stromal cells (hAAT-MSCs) or MSCs protective effects were noticeable at two weeks (one week after MSC infusions). Scale bar = 2mm.

Table S1. A summary table of the experiment outcomes. Human alpha-1 antitrypsin-overexpressed mesenchymal stromal cells (hAAT-MSCs) showed trends of better protective effects than native MSC although results did not reach significance.

	**Vehicle ± SEM**	**TNBS ± SEM**	**TNBS + MSCs ± SEM**	**TNBS + hAA-MSCs ± SEM**	**ANOVA (p-value)**	**TNBS vs. TNBS + MSCs (p-value #)**	**TNBS vs. TNBS + hAAT-MSCs (p-value #)**	**MSC vs hAAT-MSC (p-value #)**
Pancreatic area preserved (%)	93.8 ± 2.4	41.3 ± 5.2	55 ± 21.0	85.5 ± 10.9	0.03	NS	NS	NS
1 month TRPV1 expression	$1.5 \times 10^6 \pm 1.3 \times 10^5$	$4.6 \times 10^6 \pm 9.7 \times 10^5$	$1.2 \times 10^6 \pm 4.3 \times 10^5$	$1.3 \times 10^6 \pm 2.5 \times 10^5$	0.0006	*	**	NS
1 month Mast Cell density	35.2 ± 11.5	217.7 ± 52.4	90.1 ± 14	95.8 ± 18.8	0.013	*	*	NS
Abdominal withdrawal Threshold, Week 3 (VFFs, g)	0.1 ± 0.02	0.03 ± 0.004	0.04 ± 0.009	0.09 ± 0.02	0.004	NS	*	NS

SEM = Standard error of mean, TNBS = trinitrobenzene sulfonic acid, MSCs =mesenchymal stromal cells. hAAT-MSCs: human alpha-1 antitrypsin overexpressing mesenchymal stromal cells. * p<0.05, ** p<0.01, NS = not significant, # Tukey's p-value of subgroup analysis
